# Effect of Multi-Directional Forging on the Microstructure and Mechanical Properties of β-Solidifying TiAl Alloy

**DOI:** 10.3390/ma12091381

**Published:** 2019-04-28

**Authors:** Ning Cui, Qianqian Wu, Kexiao Bi, Jin Wang, Tiewei Xu, Fantao Kong

**Affiliations:** 1 School of mechanical and automotive engineering, Qingdao University of Technology, Qingdao 266520, China; wuqq2012@126.com (Q.W.); Kexiaobiqd@sina.com (K.B.); jinwangqtech@163.com (J.W.); twxu@163.com (T.X.); 2 State Key Laboratory of Advanced Welding and Joining, Harbin Institute of Technology, Harbin 150001, China; kft@hit.edu.cn

**Keywords:** TiAl, multi-directional forging, microstructure, mechanical properties

## Abstract

This study systematically investigated the influence of multi-directional forging (MDF) on the microstructural evolution, hot deformation behavior, and tensile properties of a β-solidifying TiAl alloy. The initial lamellar microstructure of an as-cast alloy was remarkably refined and homogenized by three-step MDF. High temperatures and multi-pass deformations were conducive to the decomposition of lamellae. A crack-free billet was obtained through three-step MDF, with a deformation temperature of 1250 °C and a forging speed of 0.1 mm/s, indicating that MDF can be applied to β-solidifying TiAl alloys by the proper control of the alloy composition and process parameters. Microstructural observation showed that the billet mainly consists of fine and equiaxed γ grains and a small amount of β phase. The tensile properties of the multi-directional forged alloy were also significantly improved, due to microstructure refinement. The ultimate tensile strength (UTS) and elongation (*δ*) at room temperature were 689.4 MPa, and 0.83%, respectively. The alloy exhibits excellent ductility at 700 °C. When the temperature was increased to 700 °C, the UTS decreased to 556 MPa and *δ* increased to 5.98%, indicating that the alloy exhibits excellent ductility at 700 °C. As the temperature further increased to 750 °C, *δ* dramatically increased to 46.65%, indicating that the ductile-brittle transition temperature of the alloy is between 700 °C and 750 °C.

## 1. Introduction

TiAl alloys are novel, high temperature structure materials for application in the aerospace industry, owing to their low density, high strength, and excellent creep resistance [1,2]. Great progress has been made in research on alloy design [3], phase transition [4,5], deformation mechanisms [6], and processing techniques [7] of TiAl alloys in recent years. However, low room temperature ductility has always limited to the engineering applications of TiAl alloys. Thermomechanical treatment (TMT) has been proven to be an effective way to refine the microstructure and enhance the ductility of TiAl alloys. Hot upset forging is the most common TMT approach for TiAl alloys at present [8,9]. The forged pancake has the disadvantages of inhomogeneous microstructures, many residual lamellae, and low material utilization [10]. By contrast, multi-directional forging (MDF) is a typical severe plastic deformation technology for the production of metallic materials, with ultra-fine grains, which has been successfully used in Ti alloys, Mg alloys, Al alloys, and so on [11,12,13]. However, conventional (γ + α_2_) TiAl alloys generally exhibit poor hot workability, and so are unsuitable for severe plastic deformation. Thus, only a few preliminary studies have been carried out on the multi-directional deformation of TiAl alloys, which shows that the initial coarse lamellae in TiAl alloys could be further refined by changing the forging direction [14]. It should be noted that deformed TiAl alloys generally exhibit high residual stress. The residual stress of the TiAl alloy caused by electrical discharge machining can reach 259–627 MPa [15]. High residual stress is one of the main parameters affecting both the performance and lifetime of deformed alloys, which can be probed with the aid of nanoindentation [16]. An aging treatment needs to be conducted for deformed alloys before use.

Previous studies have revealed that the hot deformability of TiAl alloys can be significantly improved by introducing a β phase. The total deformation amount could exceed 80% without cracking, when β phase content reaches a certain value [17]. These so-called β-solidifying TiAl alloys have attracted special attention [18]. The improved hot workability provides a possibility for the MDF of TiAl alloys. To date, there has been no study available on the MDF of β-solidifying TiAl alloys. In this study, the influence of MDF on the hot deformation behavior, microstructural evolution and tensile properties of the β-solidifying Ti-43Al-2Cr-1.5Mn-0.2Y alloy were investigated. The microstructural homogeneity of the single- and multi-directional forged alloy were compared. Moreover, a crack-free billet of the β-solidifying TiAl alloy was fabricated by MDF.

## 2. Experimental

**Figure 1:**
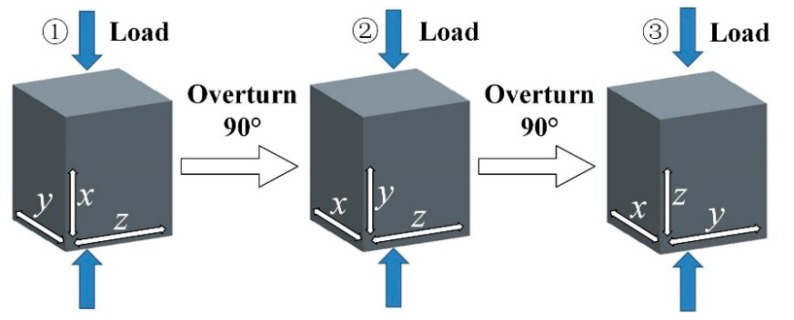
Schematic illustration of MDF.

A β-solidifying Ti-43Al-2Cr-1.5Mn-0.2Y ingot with a diameter of 90 mm and a length of 600 mm was prepared by using a consumable electrode arc melting furnace and was then annealed at 1200 °C for 4 h, in order to reduce the compositional segregation. X-ray fluorescence spectrometry (XRF, PANalytical, Almelo, Overijssel, Netherlands) revealed that the actual composition of the ingot was Ti-43.2Al-1.92Cr-1.53Mn-0.18Y. Seven small specimens (F1–F7), with dimensions of Φ 60 × 85 mm were cut from the initial ingot. All ingots were pre-heated for 1 h before deformation. An ingot (F1) was single-directional forged to a reduction of 60% at 1250 °C. Three ingots were multi-directional forged at 1250 °C (F2: One pass, F3: Two passes, F4: Three passes). The other three ingots were multi-directional forged at different temperature (F5: 1150 °C, F6: 1200 °C, F7: 1250 °C). The forging speed was 0.1 mm/s. The schematic illustration of MDF is illustrated in Figure 1. Subsequently, all samples, after forging, were transferred to a furnace at 900 °C and then cooled to room temperature with a furnace. A reheating process between different passes is not needed during MDF.

Microstructures of samples before, and after, forging were characterized using scanning electron microscopy (SEM) in backscattered electron (BSE) mode, electron backscattered diffraction (EBSD) techniques, and transmission electron microscope (TEM). The phase constituent was examined by X-ray diffraction (XRD, PANalytical, Almelo, Overijssel, Netherlands). Nano-indentation testing was conducted using Nano Indenter G200 (Agilent, Santa Clara, CA, USA). The specimens for SEM, EBSD, and TEM examinations were prepared by the mechanical polishing and the standard electro-polishing procedure, using a solution of 6% perchloric acid, 34% butanol, and 60% methanol at –20 °C at 25 V. Tensile tests were conducted on universal test machine (Instron, Boston, MA, USA) at room and elevated temperatures. SEM, EBSD, and TEM examinations were performed on a Quanta 200FEG (FEI, Hillsboro, OR, USA) equipped with an EBSD system (FEI, Hillsboro, OR, USA) and Tecnai G2 F30 (FEI, Hillsboro, OR, USA), respectively.

## 3. Results and Discussion

### 3.1. Initial As-Cast Microstructure

**Figure 2:**
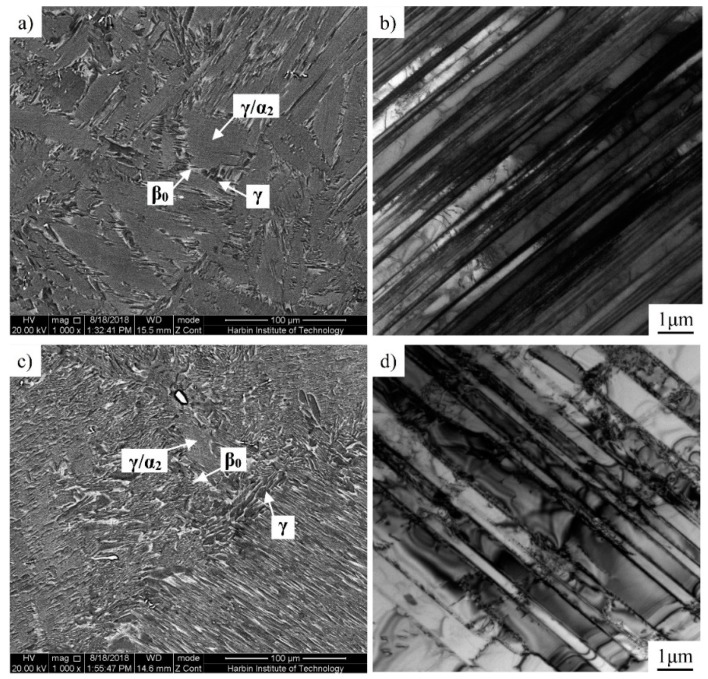
Microstructures of the as-cast Ti-43Al-2Cr-1.5Mn-0.2Y alloy. (**a**,**b**) initial condition, and (**c**,**d**) after homogenization treatment.

Figure 2 shows the microstructures of the Ti-43Al-2Cr-1.5Mn-0.2Y ingot, before and after the homogenization treatment. As shown in Figure 2a, the initial as-cast microstructure consists of a near lamellar structure, with little γ phase (black contrast) and residual β phase (grey-white contrast), presenting at the colony boundaries and triple junctions. The precipitation of β phase is attributed to the addition of the β-stabilizers Cr and Mn. It should be noted that the β phase is a disordered phase at high temperature, which exists as an ordered β0 phase at room temperature. For convenience, both disordered β and ordered β0 are denoted by β, here. The average colony size of the as-cast alloy is 50–100 μm, which is far less than that (100–300 μm) in conventional TiAl alloys [19]. The smaller colony size is ascribed to β solidification during cooling, which avoided the peritectic transformation [20]. Fine and straight lamellae were further identified by TEM, as shown in Figure 2b. According to the Ti-Al phase diagram, the solidification pathway of β solidifying TiAl alloys is L→L + β→β→β + α→α + βm→α + γ + βm→α + γ + β0→α2 + γ + β0 [21]. When the primary phase is β, twelve α variants with different orientations can be generated during the β→α phase transformation [22], which is the key reason for the microstructure refinement [23,24]. As can be seen from Figure 2c,d, the homogenized alloy still retains the features of the lamellar structure. However, the homogenization treatment led to the coarsening of lamellae. Image analysis software showed that the volume fraction of the β phase in the homogenized alloy was about 5-8%, which significantly improves the hot deformability of Ti-43Al-2Cr-1.5Mn-0.2Y alloy.

### 3.2. The Microstructure of the Single-Directional Forged Tial Alloy

**Figure 3:**
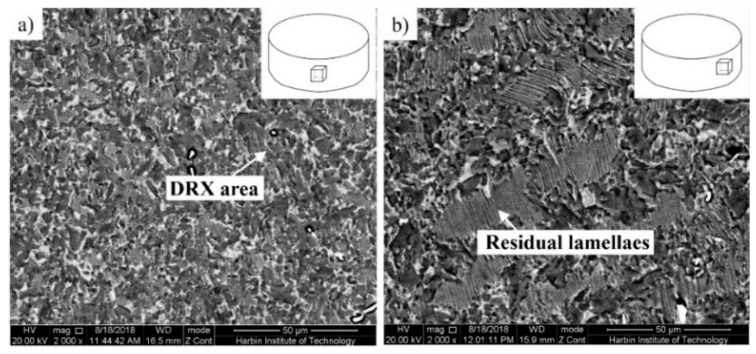
Microstructures of Ti-43Al-2Cr-1.5Mn-0.2Y alloy fabricated by SDF with a reduction of 60%. (**a**) Center area, (**b**) Bulging area.

To compare and analyze the influence of different forging methods on the microstructural evolution of the β-solidifying TiAl alloy, single-directional forging (SDF) and MDF were conducted on β-solidifying Ti-43Al-2Cr-1.5Mn-0.2Y alloy, respectively. The microstructure of the single-directional forged TiAl pancake, with a reduction of 60%, is shown in Figure 3. The microstructure at the center area (Figure 3a) is mainly composed of fine and uniform grains, indicating that the coarse lamellar microstructure had almost transformed into fine equiaxial grains. According to previous studies [9,25], it is well-known that fine equiaxial grains mainly resulted from dynamical recrystallization (DRX) during forging. As shown in Figure 3b, a large amount of residual lamellaes and some equiaxial grains can be identified in the bulging area of the pancake. Obviously, the microstructure of the pancake produced by SDF is inhomogeneous, which is related to different regions corresponding to different deformation amounts. The center area has the largest deformation, whereas the deformation in the bulging area is small. Moreover, the contact area between the billet and the anvil block is the “hard deformation zone” or “dead zone” [17]. With increases in deformation degree, the recrystallized grain size decrease and the recrystallization volume percent increases, which results in the inhomogeneous microstructure of the pancake. Moreover, it should be noted that an inhomogeneous microstructure is detrimental to the mechanical properties of the alloy, leading to low material utilization and high manufacturing cost. Thus, SDF is not an ideal method to produce TiAl billets.

### 3.3. The Microstructure of the Multi-Directional Forged Tial Alloy

**Figure 4:**
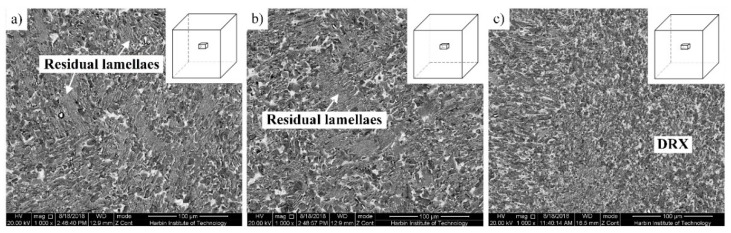
Effect of forging temperature on microstructures of Ti-43Al-2Cr-1.5Mn-0.2Y alloy. (**a**) 1150 °C, (**b**) 1200 °C, (**c**) 1250 °C.

MDF is an attractive method for preparing fine-grained metals. Temperature is an important parameter to guarantee the quality of the forged billets. It is necessary to study the effect of temperature on MDF of TiAl alloys. Figure 4 shows microstructures of the β-solidifying TiAl alloy forged at different temperatures. As shown in Figure 4a, the microstructure of the sample after three passes forging at 1150 °C indicates that some fine grains formed, and coarse lamellar colonies still remained. With the deformation temperature increasing from 1150 °C to 1200 °C, more recrystallized grains formed, and the DRX process was still incomplete, as shown in Figure 4b. The microstructure mainly consisted of coarse lamellar microstructures, surrounded by many fine grains, but the volume fraction of coarse lamellae was high. According to our experiments, when the initial temperature is lower than 1200 °C, serious cracking would occur, due to the inevitable temperature drop during forging. When the temperature further increased to 1250 °C, almost all of initial lamellae were transformed into fine grains after three passes, as shown in Figure 4c. Thus, the optimal initial forging temperature for β-solidifying Ti-43Al-2Cr-1.5Mn-0.2Y alloy should be 1250 °C or higher.

**Figure 5:**
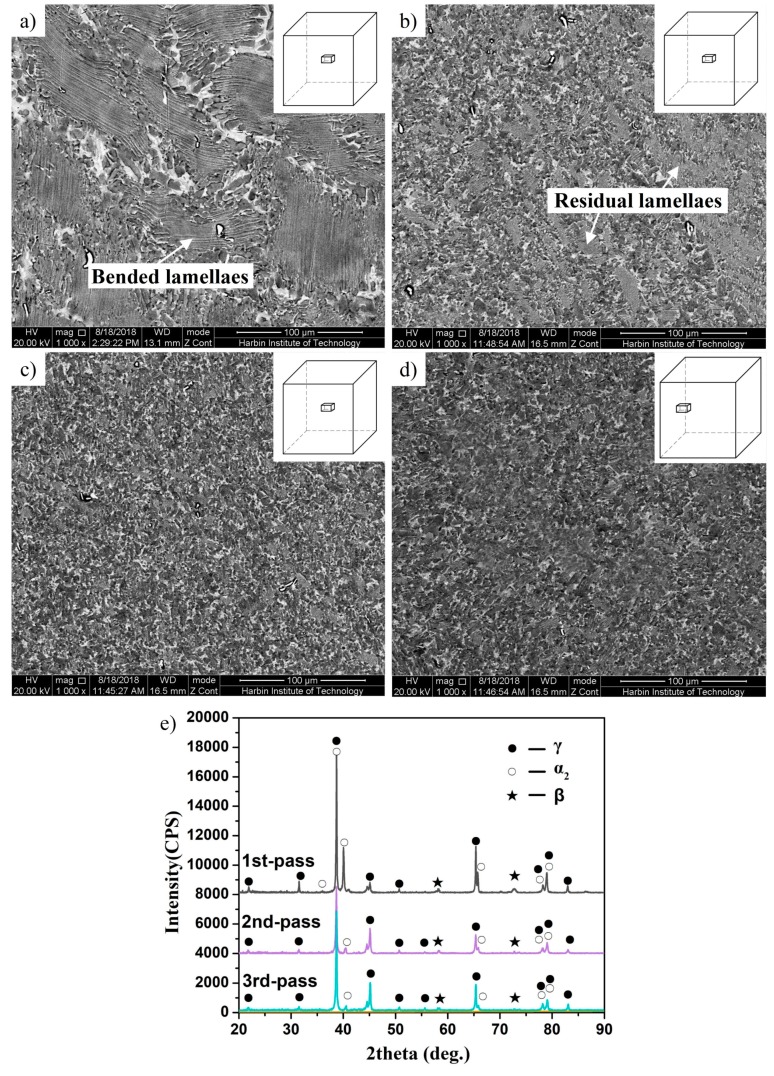
Effect of forging pass (30% reduction per pass) on microstructures of Ti-43Al-2Cr-1.5Mn-0.2Y alloy. (**a**) One pass/center area, (**b**) two passes/center area, (**c**) three passes/center area, and (**d**) three passes/bulging area. (**e**) X-ray diffraction (XRD) patterns after different forging cycles.

Figure 5 shows the microstructural evolution of the Ti-43Al-2Cr-1.5Mn-0.2Y alloy during MDF. As shown in Figure 5a, the microstructure of the alloy, after the first pass of forging, is mainly composed of bent lamellae. Nearly no equiaxed grains formed, due to the small deformation. When the alloy was deformed through the second pass of forging, it can be seen from Figure 5b that the volume fraction of equiaxed grains significantly increased, while a great quantity of residual lamellae still existed. Apparently, more deformation was needed to further refine the microstructure. The microstructures of the alloy after the third pass of forging are shown in Figure 5c,d. Figure 5c shows the deformed microstructure in the center area of the billet, which is almost completely composed of fine and equiaxed grains. This indicates that most of the initial lamellae were transformed into equiaxed grains. The similar microstructure was also observed in the bulging area, as indicated in Figure 5d. Compared with the single directional forged alloy, the microstructural homogeneity of the TiAl alloy was dramatically improved by MDF, thereby increasing material utilization and reducing the production cost. Figure 5e shows the XRD data after different forging cycles. It can be observed that the evolution of the phases is consistent with the microstructural observation.

**Figure 6:**
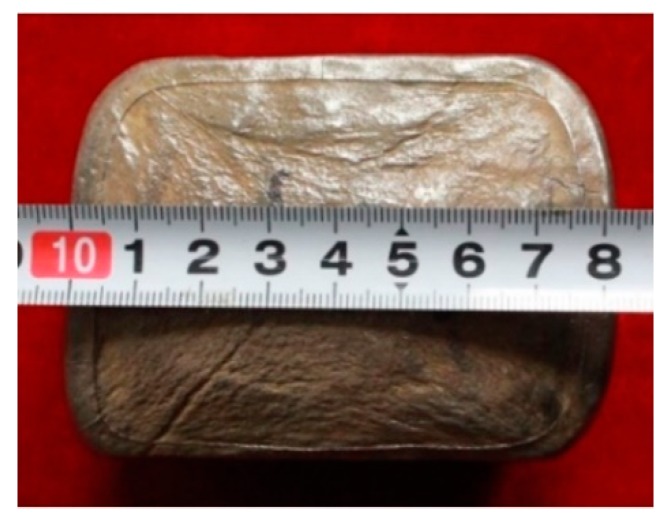
External appearance of Ti-43Al-2Cr-1.5Mn-0.2Y alloy fabricated by MDF with a reduction of 30% per pass.

To ensure the microstructural homogeneity and increase in material utilization, a crack-free TiAl billet was produced by a three-step MDF, with a reduction of 30% per pass. The initial pre-heating temperature and the forging speed are 1250 °C, and 0.1 mm/s, respectively. Figure 6 shows the external appearance of the forged TiAl billet. It can be seen that the billet exhibits a crack-free appearance. Previous studies suggested that the severe deformation is not suited for conventional γ + α_2_ TiAl alloys and high Nb-TiAl alloys, due to their poor hot workability. The deformation of these alloys are generally realized through a three-step canned SDF to avoid cracking [18,26]. Even so, cracking and residual lamellae still occur fairly often. By contrast, the β-solidifying Ti-43Al-2Cr-1.5Mn-0.2Y alloy exhibited excellent hot workability, due to the introduction of β phase, which provides a guarantee for MDF [27]. The crack-free billet suggests that MDF can be feasible in the thermomechanical treatment of β-solidifying TiAl alloys, by controlling the alloy composition and process parameters, such as β phase content, deformation temperature, and compress velocity. According to our experimental results, serious cracking of the TiAl billet occurred when the initial pre-heating temperature was lower than 1200 °C and the compress velocity was higher than 0.2 mm/s.

**Figure 7:**
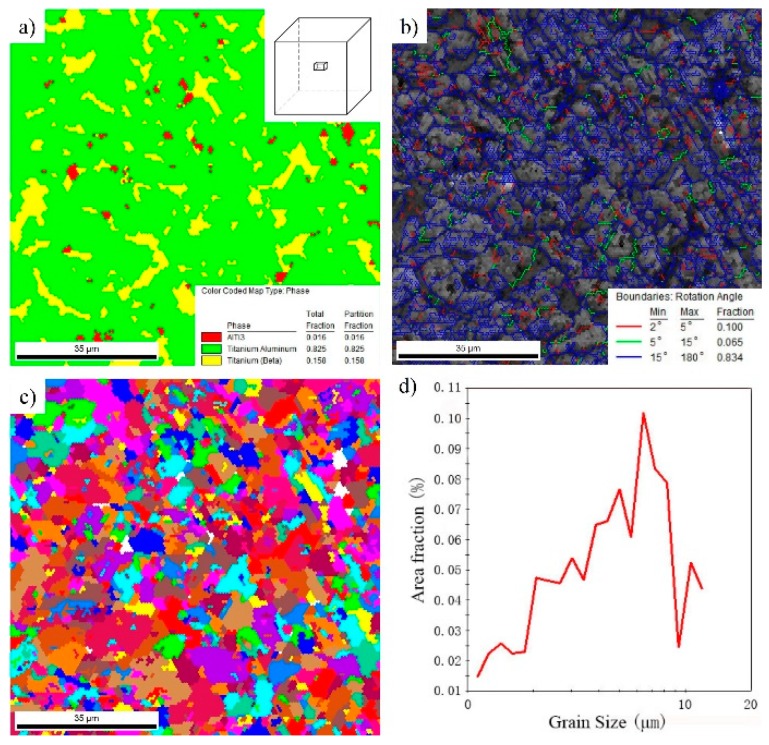
Electron back-scattered diffraction (EBSD) maps showing the microstructure of Ti-43Al-2Cr-1.5Mn-0.2Y alloy produced in three-steps forging. (**a**) Phase composition, (**b**) grain boundary characteristic, (**c**) grain map, and (**d**) grain size distribution.

In order to further analyze the deformed microstructure, the microstructures of Ti-43Al-2Cr-1.5Mn-0.2Y alloy, produced by three-step forging, were characterized using EBSD technology. Figure 7 shows the EBSD maps of the microstructure in the center area of the TiAl billet. The phase composition map, shown in Figure 7a, shows that the deformed microstructure mainly consists of 82.5% γ phase, 15.8% β phase, and 0.016% α_2_ phase. As described above, the initial as-cast microstructure is mainly characterized by γ/α_2_ lamellae, which generally contain an amount of 20 vol% α_2_ phase [28]. Obviously, most α_2_ phases are transformed into γ phase during MDF. Moreover, the content of the β phase also increased with the deformation. Figure 7b shows the distribution of the grain misorientation angle, which indicates that the low angle grain boundaries (LAGB, 2–15°) and high angle grain boundaries (HAGB, 15–180°) in the deformed microstructure are 16.5%, and 83.4%, respectively. The high density of HAGB further confirms that a large amount of recrystallized γ grains were formed by α→γ transformation. The grain map (Figure 7c) indicates that the alloy is mainly composed of uniform fine grains. The grain size distribution (Figure 7d) shows that nearly all of the grain sizes are below 10 μm, indicating that MDF can dramatically refine and homogenize the coarse microstructure of as-cast TiAl alloys.

**Figure 8:**
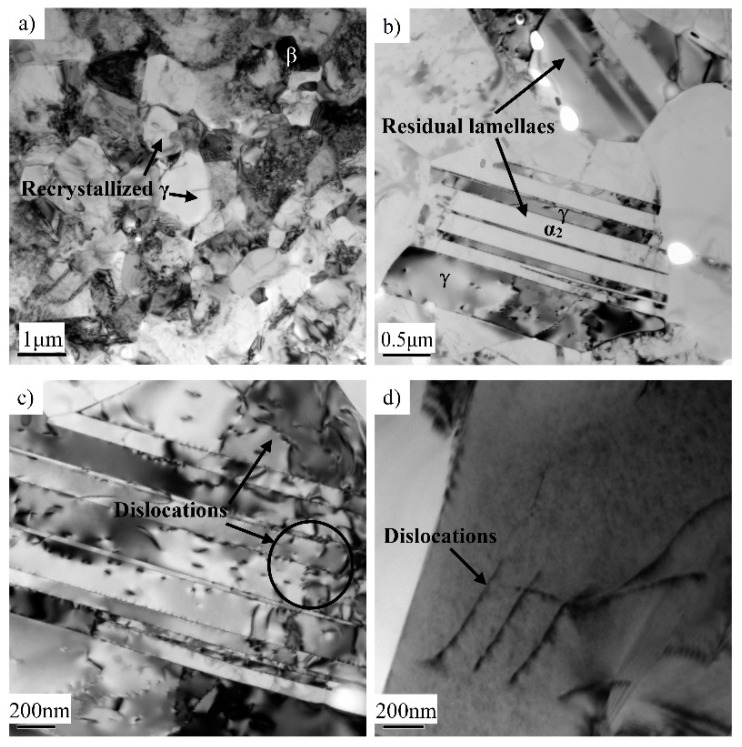
Transmission electron microscope (TEM) images showing the microstructures of Ti-43Al-2Cr-1.5Mn-0.2Y alloy after third pass forging. (**a**) DRX region, (**b**) residual lamellae, and (**c**,**d**) high-density dislocation in the γ phase

TEM investigations were conducted on the multi-directional forged specimen to study the deformation mechanism. A typical region of equiaxed grains is shown in Figure 8a, which is the main feature of the as-forged microstructure. Selected area electron diffraction confirmed that the globular grains are γ phase and the irregular grains are β phase. The deformation mechanism is closely related to the stacking fault energy (SFC). Generally, the γ phase with face-centered-cubic (fcc) structure has low SFC (60–90 mJ/m^2^) [29,30]. The SFC of the γ phase can be further decreased by the addition of Cr and Mn [31,32]. According to the literature [33], the SFC of γ phase in the Mn-containing TiAl alloy can be decreased to 52 mJ/m^2^. Thus, DRX is the main deformation mechanism of the γ phase. By contrast, the β phase, with bcc structure, exhibits low SFC and dynamic recovery is prone to occur for the β phase. Moreover, although SEM observation showed that most of initial lamellae were transformed into fine grains, a few residual lamellae were also observed using TEM (see Figure 8b) which is mainly ascribed to the lamellar orientation. The deformation amount of the lamellar microstructure depends closely on the angle (*θ*) between the uniaxial stress axis and the lamellar boundaries. When the colonies are in the soft orientation (i.e., where *θ* is about 45°), plastic deformation occurs more easily, thereby leading to fully DRX. On the contrary, the deformation amount of the colonies in hard orientations is relatively small. The lamellar colony is very difficult to decompose completely. It can also be found that the residual lamellar colonies displayed a high density of dislocations, as depicted in Figure 8c,d. High densities of dislocations provide the major driving force for the microstructural evolution of the TiAl alloy.

### 3.4. Tensile Properties and the Hardness of the Multi-Directional Forged Tial Billet

**Figure 9:**
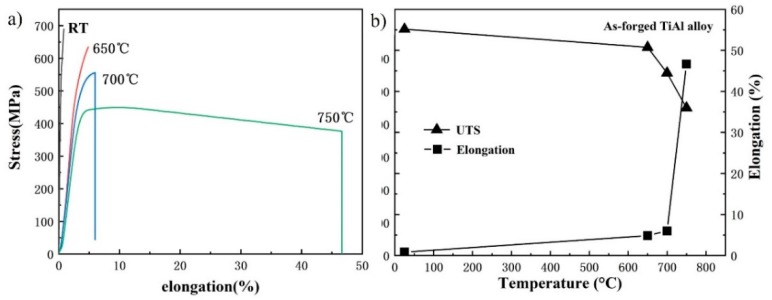
Tensile properties of the multi-directional forged TiAl alloy. (**a**) Tensile curves, and (**b**) variation of ultimate tensile strength (UTS) and *δ* with temperature.

The tensile properties of as-cast TiAl alloys are poor, due to their coarse lamellar microstructure. The room-temperature tensile strength of a TiAl ingot is generally below 500 MPa. In order to study the effect of multi-directional forging on the tensile properties of the β-solidifying TiAl alloy, tensile tests were conducted on the as-forged alloy, at different temperatures, with a constant strain rate of 1×10^−4^ s^−1^. Tensile stress-strain curves of the multi-directional forged Ti-43Al-2Cr-1.5Mn-0.2Y alloy are shown in Figure 9a. The ultimate tensile strength (UTS) and elongation (*δ*) of the as-forged alloy at room temperature are 689.4 MPa, and 0.83%, respectively. Obviously, the tensile property was significantly improved by MDF, mainly benefitting from the microstructural refinement and homogenization. Moreover, the tensile properties of multi-directional forged TiAl alloy are also sensitive to temperature. The UTS of the alloy at 650 °C is 634.1 MPa, and *δ* is 4.85%. When the temperature increased to 700 °C, the UTS decreased to 556 MPa and *δ* increased to 5.98%. Moreover, as shown in Figure 9b, it can be found that the UTS decreased to 449.7 MPa and *δ* dramatically increased to 46.65% when the deformation temperature further increased to 750 °C. It can be concluded that the ductile-brittle transition temperature (DBTT) of the multi-directional forged Ti-43Al-2Cr-1.5Mn-0.2Y alloy is between 700 °C and 750 °C. Thus, the Ti-43Al-2Cr-1.5Mn-0.2Y alloy is generally suitable for high temperatures below 700 °C. Moreover, nano-indentation testing was also conducted on the multi-directional forged alloy. Three indents were performed on the alloy to obtain the average value and maintain accurate measurement results. The result shows that the hardness and the elastic modulus of the alloy are 4.37 GPa, and 171.3 GPa, respectively.

## 4. Summary

(1) A crack-free billet of β-solidifying Ti-43Al-2Cr-1.5Mn-0.2Y alloy was fabricated successfully by MDF. This has proved that severe plastic deformation, such as MDF, can be used to produce high-quality β-solidifying TiAl billets, by controlling the β phase content, deformation temperature, and compress velocity.

(2) The multi-directional forged alloy exhibits a fine and uniform microstructure, which is mainly composed of an equiaxial γ phase and irregular β phase. Most of the initial as-cast γ/α_2_ lamellae were transformed into equiaxial γ grains by DRX. Compared with SDF, MDF can further refine the microstructure and improve the microstructural homogeneity, thereby enhancing material utilization.

(3) The multi-directional forged alloy exhibits excellent tensile properties. The UTS and *δ* at room temperature are 689.4 MPa, and 0.83%, respectively. When the temperature increased to 700 °C, the UTS and *δ* can reach to 556 MPa, and 5.98%, respectively. The value of *δ* increased dramatically to 46.65% when the temperature was further increased to 750 °C. The ductile-brittle transition temperature of the multi-directional forged alloy was, therefore, determined to be between 700 °C and 750 °C.
